# Association of residual ductal carcinoma in situ with breast cancer treatment outcomes after neoadjuvant chemotherapy according to hormone receptor status

**DOI:** 10.1007/s12672-024-01157-z

**Published:** 2024-07-17

**Authors:** Eunju Shin, Tae-Kyung Yoo, Jisun Kim, Il Yong Chung, Beom Seok Ko, Hee Jeong Kim, Jong Won Lee, Byung Ho Son, Sae Byul Lee

**Affiliations:** Division of Breast Surgery, Department of Surgery, University of Ulsan College of Medicine, Asan Medical Center, 88, Olympic-Ro 43-Gil, Songpa-Gu, Seoul, 05505 Korea

**Keywords:** Pathologic complete response, Residual ductal carcinoma in situ, Neoadjuvant chemotherapy, Breast cancer, Prognosis, Hormone receptor

## Abstract

**Purpose:**

This research aimed to clarify the impact of residual ductal carcinoma in situ(DCIS) in surgical specimens obtained after neoadjuvant chemotherapy(NAC) for breast cancer on the associated prognosis outcomes.

**Methods:**

This retrospective study was performed on a cohort of 1,009 patients who achieved pCR following NAC for breast cancer and underwent subsequent breast surgery at a single institution between January 2008 and December 2019. Overall survival, local recurrence-free survival, distant metastasis-free survival, and disease-free survival of the residual and non-residual DCIS groups were the outcomes compared, with further subgroup analysis performed according to hormone receptor status.

**Results:**

260 individuals (25.8%) presented with residual DCIS. Based on a median follow-up of 54.0 months, no significant differences in outcomes were observed between the two groups. Patients with residual DCIS and hormone receptor-negative (HR-) breast cancer demonstrated a significant decrease in distant metastasis-free survival (*p* = 0.030) compared to those without residual DCIS. In the HR + cohort, no significant difference was observed between the two groups. Multivariate analysis of the HR- cohort demonstrated a significant association between residual DCIS and an elevated risk for distant recurrence (hazard ratio = 2.3, 95% confidence interval = 1.01–5.20, *p* = 0.047).

**Conclusions:**

Residual DCIS following NAC may impact breast cancer outcomes, particularly with respect to the occurrence of distant metastasis in HR- patients. Therefore, clinicians must vigilantly monitor patients with residual DCIS after NAC, and further research is needed to expand our understanding of the clinical implications of residual DCIS.

**Supplementary Information:**

The online version contains supplementary material available at 10.1007/s12672-024-01157-z.

## Introduction

Breast cancer has become a significant health concern among women worldwide, and its treatment requires a multi-modal approach to ensure better patient outcomes [1, 2]. Among the diverse therapeutic modalities, neoadjuvant chemotherapy (NAC) has become an essential tool in managing breast cancer, particularly for patients with locally advanced disease, high-risk tumors, and early-stage cancer, as it downsizes the tumor and improves surgical and oncological outcomes [3, 4].

With the recent advances in NAC, there has been a concurrent increase in the rate of pathologic complete response (pCR), and pCR following NAC has become a critical predictor of clinical outcomes. Approximately 21% of the patients undergoing NAC achieve a pCR [1]. The association between pCR and enhanced disease-free survival (DFS) and overall survival (OS) is well known, particularly in aggressive breast cancer subtypes such as triple-negative and HER2-positive breast cancers [5–7].

However, pCR is currently defined as the absence of invasive cancer in the breast and axillary lymph nodes after NAC, which includes the presence of residual ductal carcinoma in situ (DCIS); consequently, this inclusion is the subject of ongoing debate [8]. The potential implications of residual DCIS following NAC remain contested. Some researchers suggest that residual DCIS could be a predictor of worse outcomes [9, 10], whereas others argue that there is no empirical evidence proving the adverse impact of residual DCIS on patient outcomes [2, 11, 12].

Considering this controversy, our study aims to explore the impact of residual DCIS in pCR after NAC on breast cancer outcomes compared with the outcomes in the absence of residual DCIS. Additionally, we aim to perform a subgroup analysis by biological subtype to assess the prognostic implications of residual DCIS after NAC.

## Methods

### Patients

**Fig. 1:**
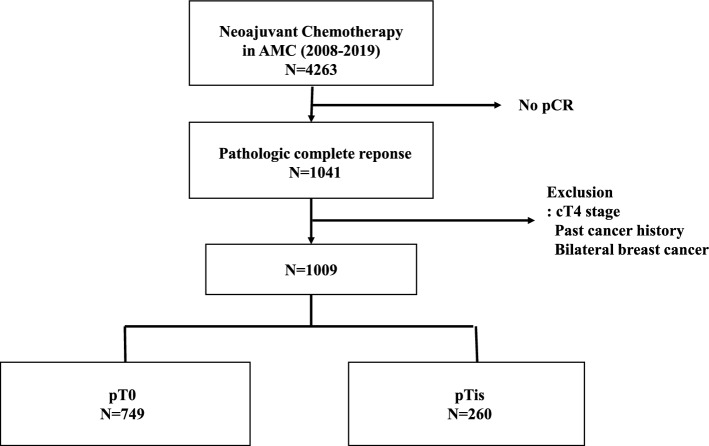
Patient flowchart

In this retrospective study, we obtained data from a cohort of 4,263 patients who underwent NAC and subsequent surgery at Asan Medical Center in Seoul, Korea, over a period spanning from January 2008 and December 2019. A total of 1,041 individuals achieved a pCR from the study cohort. The exclusion criteria were clinical stage 4 before NAC, past history of malignancy, and bilateral breast cancer. The final count of patients who achieved a pCR was 1,009 (Fig. 1). Prior to NAC, all patients underwent core needle biopsy and radiologic examination in order to assess their clinical stage. In regard to the systemic chemotherapy, most patients were treated with the Adriamycin and Taxol-based chemotherapy, and patients were treated based on the standard treatment, such as, patients had received breast conserving surgery or mastectomy and axillary operation, patients with HR + breast cancer were managed with tamoxifen or aromatase inhibitor, with or without ovarian function suppression. Also, those with HER2 + cancers received adjuvant targeted therapy and patients who had breast conserving surgery received radiotherapy. As for some patients whose pathologic results were changed after NAC, both pre-treatment and post-treatment findings were taken into consideration for adjuvant therapy. pCR was subsequently categorized into two groups: ypT0 and ypTis, based on the presence and absence of DCIS, respectively. This study was reviewed and approved by the Institutional Review Board of the Asan Medical Center (2022–0282) and informed consent was waived because the study was based on retrospective clinical data. And data were accessed for research purposes from January to February 2023.

### Pathologic assessments

The evaluation of pathology in this study is based on specimens collected at the time of definitive surgical resection after neoadjuvant chemotherapy, utilizing the Residual Cancer Burden (RCB) method [13]. This method is composite measure such as, bidimensional measurement of the primary tumor bed, overall invasive cancer cellularity, the percentage of DCIS in the primary tumor bed, the number of positive lymph nodes, and the diameter of the largest lymph node metastasis. The presence of residual DCIS was determined using the percentage of DCIS in the primary tumor bed from the RCB assessment. If the percentage was 0, it was designated as no residual DCIS present, while any percentage greater than 0 was marked as presence of residual DCIS.

### Statistical analysis

In order to assess the impact of residual DCIS following NAC, we evaluated five breast cancer outcomes, namely, the OS, local recurrence-free survival (LFS), regional recurrence-free survival (RFS), distant metastasis-free survival (DMFS), and DFS of the two categorized groups, pT0 and pTis. Then, to determine the relationship between residual DCIS and the outcomes, a subgroup analysis and multivariate analysis were performed based on the cancer subtype. LFS is the time from the date of surgery to local recurrence including recurrent carcinoma in situ and chest wall at ipsilateral breast, RFS is defined as a metastatic disease in the ipsilateral axillary, internal mammary or supraclavicular or infraclavicular nodes, with or without involvement of the ipsilateral breast tissue, and DMFS is the time from the date of surgery to distant metastasis recurrence. DFS was defined as the time from the date of surgery to the earliest local recurrence, regional recurrence, or distant metastasis and any deaths. OS was defined as the time from the date of diagnosis of breast cancer to any deaths, whether they were breast cancer-related or not.

We analyzed the baseline variables, which were stratified by the presence or absence of residual DCIS, by performing two-sided chi-squared analysis, Fisher’s exact test, and Mann–Whitney U test to determine the significance of the results. LFS, DMFS, DFS, and OS were plotted using the Kaplan–Meier product-limit method, and log-rank p-value was calculated. To assess the prognostic impact of the clinicopathologic factors, hazard ratios, 95% confidence intervals, and p-value were calculated using the Cox proportional hazards model. All statistical tests were two-tailed, and a p-value less than 0.05 was considered statistically significant. Statistical analyses were performed using the Statistical Package for the Social Sciences (SPSS, ver. 20, Armonk, NY, USA).

## Results

### Patients’ characteristics

Within the study cohort, 260 (25.8%) exhibited residual DCIS, with a median follow-up of 54.0 months (0.2–161.9 months). The study findings indicate that the median age at diagnosis was 48.2 years (20–79 years) (Table 1). A large proportion of patients were diagnosed with cT2 stage (64.3%), N1 stage (45.6%), and clinical stage II breast cancer (60.6%). The more patients were hormone receptor negative (64.3%) and HER2 negative (64.3%). Most patients had G2 histologic grade (53.9%) and G2 nuclear grade (53.7%). Strong P53 expression was found in 52.7% of the patients. High Ki-67 expression was observed in 88.8% of patients.

**Table 1:** Comparison of characteristics between pT0 versus pTis

Characteristics	Total	pT0	pTis	p-value
Age (year) (Mean ± SD)	48.28 ± 10.49	48.26 ± 10.84	48.34 ± 9.40	0.530
Breast operation				0.001
Breast conserving surgery	598 (59.3)	466 (62.2)	132 (50.8)	
Total mastectomy	411 (40.7)	283 (37.8)	128 (49.2)	
Axillary operation				0.292
No	2 (0.2)	1 (0.1)	1 (0.4)	
Sentinel node biopsy	740 (73.3)	558 (74.5)	182 (70.0)	
Axillary lymph node dissection	267 (26.5)	190 (25.4)	77 (29.6)	
cT stage				0.054
T0	5 (0.5)	5 (0.7)	0 (0.0)	
Tis	2 (0.2)	1 (0.1)	1 (0.4)	
T1	116 (11.5)	91 (12.2)	25 (9.7)	
T2	646 (64.3)	492 (65.8)	154 (59.9)	
T3	201 (20.0)	134 (17.9)	67 (26.1)	
T4	35 (3.5)	25 (3.3)	10 (3.9)	
Unknown	4	1	3	
cN stage				0.913
N0	270 (26.9)	200 (26.7)	70 (27.5)	
N1	457 (45.6)	342 (45.7)	115 (45.1)	
N2	88 (8.8)	68 (9.1)	20 (7.8)	
N3	188 (18.7)	138 (18.4)	50 (19.6)	
Unknown	6	1	5	
Clinical stage				0.360
I	17 (1.7)	12 (1.6)	5 (2.0)	
II	608 (60.6)	463 (61.9)	145 (56.9)	
III	378 (37.7)	273 (36.5)	105 (41.2)	
Unknown	6	1	5	
Hormone receptor				0.007
Negative	642 (64.3)	495 (66.7)	147 (57.4)	
Positive	356 (35.7)	247 (33.3)	109 (42.6)	
Unknown	11	7	4	
HER2 status				< 0.001
Negative	642 (64.3)	495 (66.7)	147 (57.4)	
Positive	356 (35.7)	247 (33.3)	109 (42.6)	
Unknown	11	7	4	
Histologic grade				0.033
G1	5 (0.5)	3 (0.4)	2 (0.8)	
G2	530 (53.9)	377 (51.6)	153 (60.5)	
G3	448 (45.6)	350 (47.9)	98 (38.7)	
Unknown	26	19	7	
Nuclear grade				0.032
G1	4 (0.4)	4(0.5)	0(0.0)	
G2	533 (53.7)	379(51.4)	154(60.2)	
G3	456 (45.9)	354(48.0)	102(39.8)	
Unknown	16	12	4	
P53				0.233
Negative	310 (32.2)	238 (33.2)	72 (29.0)	
Weak	73 (7.6)	49 (6.8)	24 (9.7)	
Intermediate	73 (7.6)	50 (7.0)	23 (9.3)	
Strong	508 (52.7)	379 (52.9)	129 (52.0)	
Unknown	45	33	12	
Ki-67				0.179
Low (≤ 20%)	113 (11.2)	78 (10.4)	35 (13.5)	
High (> 20%)	896 (88.8)	671 (89.6)	225 (86.5)	
pN stage				0.636
N0	883 (87.5)	661 (88.3)	222 (85.4)	
N1	103 (10.2)	71 (9.5)	32 (12.3)	
N2	15 (1.5)	11 (1.5)	4 (1.5)	
N3	8 (0.8)	6 (0.8)	2 (0.8)	
Radiotherapy				0.662
No	173 (17.2)	126(16.9)	47(18.1)	
Yes	833 (82.8)	620(83.1)	213(81.9)	
Unknown	3	3	0	
Chemotherapy				0.838
Neoadjuvant	984 (97.5)	730 (97.5)	254 (97.7)	
Neoadjuvant + Adjuvant	25 (2.5)	19 (2.5)	6 (2.3)	
Hormone therapy				< 0.001
No	622 (61.7)	494 (66.0)	128 (49.2)	
Yes	386 (38.3)	254 (34.0)	132 (50.8)	
Unknown	1	1	0	

Data shown are number (%) not otherwise specified

*SD* standard deviation, *HER2* human epidermal growth factor receptor 2, *G* grade

Subsequently, our investigation focused on the characteristics stratified by the presence or absence of residual DCIS (Table 1). Notably, variables such as the type of breast operation, hormone receptor status, HER2 status, histologic grade, nuclear grade, and hormone therapy displayed a statistically significant correlation with residual DCIS. There were significant differences in several other characteristics, including breast operation (*p* = 0.001), hormone receptor status (*p* = 0.007), HER2 status (*p* < 0.001), histologic grade (*p* = 0.033), nuclear grade (*p* = 0.032) and hormone therapy (*p* < 0.001). Specifically, patients with pTis breast cancer were more likely to undergo breast-conserving surgery than total mastectomy, and were more likely to have positive HER2 status, and lower histologic and nuclear grades compared to those with pT0 breast cancer.

### Survival outcomes according to residual DCIS

**Fig. 2:**
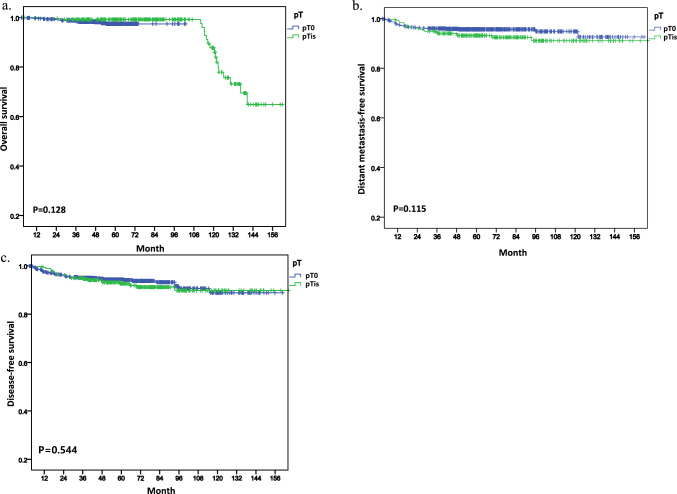
Kaplan–Meier curves showing survival outcomes in overall population

Figure 2 displays the Kaplan–Meier curves for OS, DMFS, and DFS, stratified by the presence or absence of residual DCIS within the total study population. Notably, no statistically significant differences were observed between the two groups. Among the 66 patients who exhibited recurrence, 21 events of local recurrence, 19 events of regional recurrence, and 46 events of distant metastasis were reported within a median follow-up period of 54.0 months (0.2–161.9 months). Notably, 33 patients died during this period. We also found that patients with DCIS ( +) had a 5-year OS rate of 99.2% compared to those with DCIS (−) who had an OS rate of 97.4% (*p* = 0.128) (Fig. 2a). However, the 5-year rates of DMFS and DFS were all lower in patients with DCIS ( +) compared to those with DCIS (−). Specifically, the five-year LFS rate was 76.1% for DCIS ( +) and 92.1% for patients with DCIS (−) (*p* = 0.603), while the 5-year DMFS rate was 77.6% for patients with DCIS ( +) and 92.2% for patients with DCIS (−) (*p* = 0.115). The 5-year DFS rate was 60.6% for patients with DCIS ( +) and 92.2% for patients with DCIS (−) (*p* = 0.544) (Fig. 2b, c). These findings suggest that patients with DCIS ( +) may have tendency of a higher risk of disease recurrence and should be closely monitored during follow-up.

**Fig. 3:**
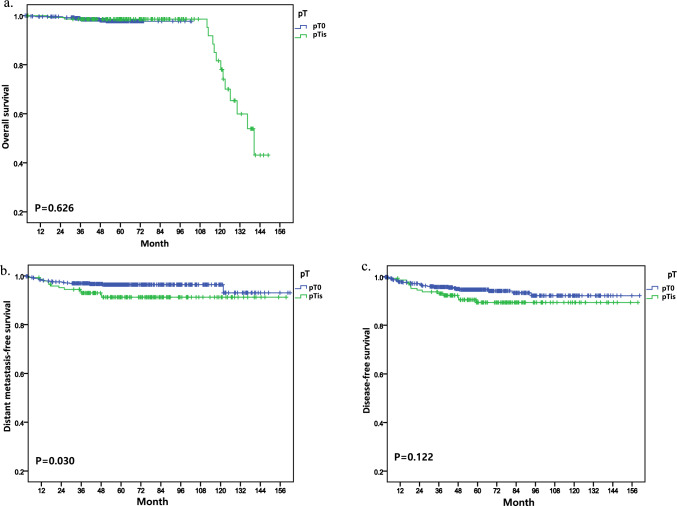
Kaplan–Meier curves showing survival outcomes in hormone receptor negative status

**Fig. 4:**
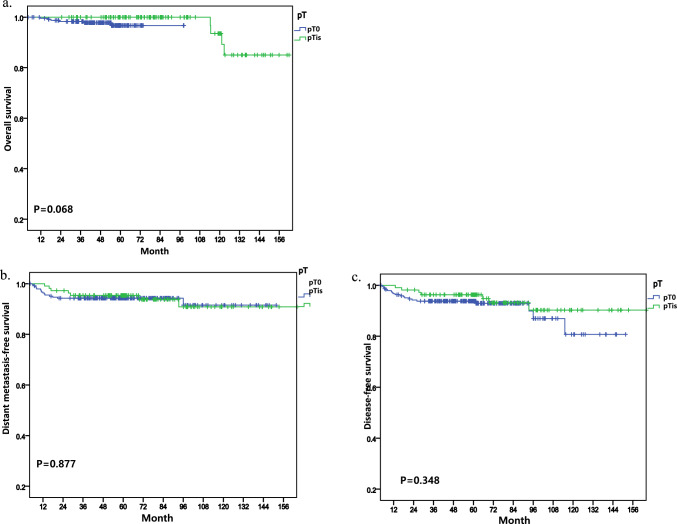
Kaplan–Meier curves showing survival outcomes in hormone receptor positive status

According to the analysis based on hormone receptor status, patients with residual DCIS and hormone receptor-negative (HR−) breast cancer demonstrated a statistically significant decrease in DMFS (*p* = 0.030) compared to those without residual DCIS (Fig. 3b) but there was not statistical significance in OS (Fig. 3a). Furthermore, although a decrease in DFS was observed, it did not reach statistical significance (*p* = 0.122) (Fig. 3c). In the cohort of patients with hormone receptor-positive (HR +) tumors, analyses did not reveal any statistically significant differences in OS, DMFS, DFS (Fig. 4).

Further investigation into the characteristics of patients with HR- cancer revealed a higher incidence of residual DCIS among those who underwent total mastectomy and presented with HER2-positive status (Table 2). Table 2 shows no significant differences in age between the two groups (*p* = 0.951). However, there were significant differences in the type of breast surgery (*p* = 0.001), with more patients in the residual DCIS group undergoing breast-conserving surgery and more patients in the non-residual DCIS group undergoing total mastectomy. There were no significant differences in cT stage (*p* = 0.092) and cN stage (*p* = 0.304) between the two groups. However, there was a trend towards a higher clinical stage in the residual DCIS group (*p* = 0.149). There was a significant difference in HER2 status between the two groups (*p* < 0.001); more patients in the residual DCIS group had HER2-positive tumors. There was also a trend towards higher histologic grade in patients with residual DCIS (*p* = 0.087) and higher nuclear grade (*p* = 0.126), although the differences did not reach statistical significance. Moreover, there was a significant difference in the number of patients who received hormone therapy (*p* < 0.001); more patients with residual DCIS received hormone therapy.

**Table 2:** Comparison of characteristics between pT0 versus pTis according to hormone receptor status

Characteristics	HR negative status			HR positive status		
	pT0	pTis	p-value	pT0	pTis	p-value
Age (year) (Mean ± SD)	48.73 ± 10.967	48.71 ± 9.61	0.951	47.24 ± 10.57	47.95 ± 9.28	0.268
Breast operation			0.001			0.642
Breast conserving surgery	322 (65.1)	73 (49.7)		138 (55.9)	58 (53.2)	
Total mastectomy	173 (34.9)	74 (50.3)		109 (44.1)	51 (46.8)	
Axillary operation			0.274			0.401
No	1 (0.2)	1 (0.7)		0 (0.0)	0 (0.0)	
Sentinel node biopsy	369 (74.5)	101 (68.7)		185 (74.9)	77 (70.6)	
Axillary lymph node dissection	125 (25.3)	45 (30.6)		62 (25.1)	32 (29.4)	
cT stage			0.092			0.337
T0	3 (0.6)	0 (0.0)		1 (0.4)	0 (0.0)	
Tis	0 (0.0)	1 (0.7)		1 (0.4)	0 (0.0)	
T1	58 (11.7)	14 (9.6)		33 (13.4)	10 (9.3)	
T2	323 (65.3)	85 (58.2)		165 (67.1)	67 (62.6)	
T3	95 (19.2)	40 (27.4)		37 (15.0)	26 (24.3)	
T4	16 (3.2)	6 (4.2)		9 (3.7)	4 (3.7)	
Unknown	0	1		1	2	
cN stage			0.304			0.165
N0	141 (28.5)	39 (26.7)		55 (22.4)	29 (27.6)	
N1	217 (43.8)	55 (37.7)		123 (50.0)	58 (55.2)	
N2	45 (9.1)	16 (11.0)		22 (8.9)	4 (3.8)	
N3	92 (18.6)	36 (24.7)		46 (18.7)	14 (13.3)	
Unknown	0	1		1	4	
Clinical stage			0.149			0.774
I	7 (1.4)	3 (2.1)		5 (2.0)	1 (1.0)	
II	302 (61.0)	76 (52.1)		155 (63.0)	67 (63.8)	
III	186 (37.6)	67 (45.9)		86 (35.0)	37 (35.2)	
Unknown	0	1		1	4	
HER2 status			< 0.001			0.541
Negative	255 (51.7)	49 (33.3)		101 (41.1)	41 (37.6)	
Positive	238 (48.3)	98 (66.7)		145 (58.9)	68 (62.4)	
Unknown	2	0		1	0	
Histologic grade			0.087			0.700
G1	1 (0.2)	1 (0.7)		2 (0.8)	1 (0.9)	
G2	237 (48.8)	83 (58.0)		139 (57.9)	67 (62.6)	
G3	248 (51.0)	59 (41.3)		99 (41.3)	39 (36.4)	
Unknown	9	4		7	2	
Nuclear grade			0.126			0.454
G1	2 (0.4)	0 (0.0)		2 (0.8)	0 (0.0)	
G2	237 (48.5)	84 (57.5)		141 (57.8)	68 (63.0)	
G3	250 (51.1)	62 (42.5)		101 (41.4)	40 (37.0)	
Unknown	6	1		3	1	
P53			0.442			0.756
Negative	163 (34.0)	40 (28.2)		74 (31.8)	31 (29.5)	
Weak	14 (2.9)	7 (4.9)		35 (14.8)	17 (16.2)	
Intermediate	25 (5.2)	8 (5.6)		25 (10.6)	15 (14.3)	
Strong	278 (57.9)	87 (61.3)		101 (42.8)	42 (40.0)	
Unknown	15	5		11	4	
Ki-67			0.253			0.699
Low (≤ 20%)	39 (7.9)	16 (10.9)		39 (15.8)	19 (17.4)	
High (> 20%)	456 (92.1)	131 (89.1)		208 (84.2)	90 (82.6)	
pN			0.393			0.956
N0	442 (89.3)	124 (84.4)		214 (86.6)	95 (87.5)	
N1	45 (9.1)	20 (13.6)		24 (9.7)	11 (10.1)	
N2	4 (0.8)	2 (1.4)		7 (2.8)	2 (1.8)	
N3	4 (0.8)	1 (0.7)		2 (0.8)	1 (0.9)	
Radiotherapy			0.299			0.441
No	70 (14.2)	26 (17.7)		54 (22.0)	20 (18.3)	
Yes	423 (85.8)	121 (82.3)		192 (78.0)	89 (81.7)	
Unknown	2	0		1	0	
Chemotherapy			0.330			0.151
Neoadjuvant	485 (98.0)	142 (96.6)		238 (96.4)	108 (99.1)	
Neoadjuvant + Adjuvant	10 (2.0)	5 (3.4)		9 (3.6)	1 (0.9)	
Hormone therapy			< 0.001			0.054
No	451 (91.1)	116 (78.9)		39 (15.9)	9 (8.3)	
Yes	44 (8.9)	31 (21.1)		207 (84.1)	100 (91.7)	
Unknown	0	0		1	0	

Data shown are number (%) not otherwise specified

*SD* standard deviation, *HER2* human epidermal growth factor receptor 2, *G* grade

According to the univariate analysis of the HR- cohort, age was not statistically significant (*p* = 0.166), but cN stage (hazard ratio = 3.42, 95% confidence interval = 0.99–11.83, *p* = 0.046) and residual DCIS (hazard ratio = 2.21, 95% confidence interval = 1.06–4.58, *p* = 0.034) were statistically significant worse predictors of the DMFS (Table 3). Other variables, such as operation, cT, histologic grade, nuclear grade, p53, Ki-67, HER2 status, and radiotherapy were not statistically significant. Based on our multivariate analysis of the HR- cohort, residual DCIS remained a detrimental factor for DMFS (hazard ratio = 2.46, 95% confidence interval = 1.01–5.98, *p* = 0.047). However, in patients with hormone receptor-positive (HR +) cancer, our analysis did not reveal any significant associations with the outcomes of interest.

**Table 3:** Univariable and multivariable regression analysis in DMFS of hormone receptor negative patients

Variables	Univariable		Multivariable	
	HR (95% CI)	P-value	HR (95% CI)	P-value
Age	0.98 (0.94–1.01)	0.166	0.96 (0.92–1.01)	0.095
Operation		0.538		0.320
Breast conserving surgery	1 (Ref)		1 (Ref)	
Total Mastectomy	1.26 (0.61–2.59)		0.55 (0.19–1.54)	
cT		0.418		0.924
cT0-1	1 (Ref)		1 (Ref)	
cT2-4	0.67 (0.26–1.76)		0.96 (0.27–3.40)	
cN		0.046		0.191
cN0	1 (Ref)		1(Ref)	
cN1	3.42 (0.99–11.83)		3.15 (0.87–11.38)	
cN2	4.22 (0.95–18.88)		3.08 (0.49–19.54)	
cN3	3.86 (1.02–14.54)		5.81 (1.38–24.46)	
HER2 status	1.09 (0.53–2.23)	0.819	1.05 (0.44–2.49)	0.806
Histologic grade		0.188		0.900
G1-2	1 (Ref)		1 (Ref)	
G3	0.60 (0.28–1.28)		1.27 (0.04–40.94)	
Nuclear grade		0.173		0.668
G1-2	1 (Ref)		1 (Ref)	
G3	0.59 (0.28–1.26)		0.48 (0.02–15.38)	
p53		0.081		0.256
Negative	1 (Ref)		1 (Ref)	
Weak	2.30 (0.65–8.08)		0.59 (0.07–4.84)	
Intermediate	0.99 (0.22–4.39)		1.15 (0.24–5.42)	
Strong	0.50 (0.23–1.09)		0.41 (0.17–1.01)	
Ki-67	1.00 (0.98–1.01)	0.703	1.00 (0.98–1.02)	0.826
Radiotherapy	1.51 (0.46–4.98)	0.502	1.09 (0.22–5.47)	0.918
Residual DCIS	2.21 (1.06–4.58)	0.034	2.46 (1.01–5.98)	0.047

*HER2* human epidermal growth factor receptor 2, *G* grade

## Discussion

Currently, NAC is gaining importance in the treatment of not only advanced breast cancer but also early-stage breast cancer. This increase in importance is attributed to the surge in the rate of pCR. Numerous studies that examine the relationship between pCR rates and breast cancer treatment outcomes are being conducted. According to the CTNeoBC pooled analysis of 12 identified international trials with 11,955 patients, pCR was associated with improved DFS (ypT0ypN0: hazard ratio = 0.44, 95% confidence interval = 0.39–0.51; ypT0/isypN0: hazard ratio = 0.48, 95% confidence interval = 0.43–0.54) and OS (ypT0ypN0: hazard ratio = 0.36, 95% confidence interval = 0.30–0.44; ypT0/isypN0: hazard ratio = 0.36, 95% confidence interval = 0.45–0.42) [9]. In this study, the attainment of ypT0N0 after NAC in the overall population was associated with significantly enhanced DFS, consistent with numerous studies. In contrast, another study that compared patients who achieved ypT0N0 with those who did not indicate an increased hazard ratio for DFS when residual DCIS was present [10].

Considering the poor prognosis of residual DCIS indicated in this study, it is imperative to define pCR, particularly in relation to the presence of DCIS. Therefore, the role of residual DCIS in predicting prognosis after NAC in breast cancer patients was investigated in this study. Within this study, the rate of residual DCIS in patients who achieved pCR was observed to be 10.7%. This rate aligns with the rates calculated in other studies, which range between approximately 10% and 20%. For example, in an I-SPY2 trial of 337 patients without residual invasive disease, 70 (21%) had residual DCIS [2]. In another study, Ploumen et al. [1] found that 881 (13.1%) patients had residual DCIS. The prevalence of residual DCIS is of a magnitude that cannot be overlooked when defining pCR. In other study that focused on the definition of pCR (pT0 or pT0/is), Choi et al. observed that patients with ypT0/is who achieved pCR demonstrated better prognosis in terms of DMFS and OS than those who did not achieve pCR. Interestingly, the exclusion of DCIS from the definition of pCR did not statistically impact the OS [5]. This finding provides an additional perspective over the inclusion of DCIS in the definition of pCR.

A limited number of studies evaluate breast cancer treatment outcomes after NAC by comparing the presence or absence of residual DCIS in cases of pCR. One study assessed the DFS and OS of patients who achieved pCR and demonstrated an increased risk in the residual DCIS group by exhibiting a hazard ratio of 1.74 (95% confidence interval = 1.28–2.36, *p* < 0.001), compared to pCR in the non-residual DCIS group [10]. In our research, a decrease in DFS was also identified, although it did not reach statistical significance.

Moreover, it is well-established that the rate of pCR varies across different breast cancer subtypes, with the lowest pCR rate observed in luminal A subtype and the highest in HER2 and triple-negative breast cancer (TNBC) subtypes. Given that the molecular subtype serves as an independent prognostic predictor for both pCR and OS, it is worth noting that clinical downstaging is linked with enhanced survival outcomes, particularly in cohorts with HER2-positive and TNBC subtypes [14]. In this study, the rates of pCR observed in each subtype were as follows: 14.3%, 21.4%, 33.8%, and 30.6% for luminal A, luminal B, HER2, and TNBC subtypes, respectively. Additionally, the presence of residual DCIS was highest in patients with HER2 subtype, constituting 38.3% of cases. Consequently, we performed a subgroup analysis according to the hormone receptor status by dividing the sample population into HR + and HR- subgroups. In the HR- subgroup, a decline in DMFS and DFS was observed in cases with residual DCIS; only the difference in DMFS reached statistical significance. This result allows us to postulate two possible hypotheses. Firstly, when comparing ypT0N0 to ypTisN0, the latter implies the remaining of cancer components in the breast after NAC, which could be interpreted as a suboptimal response to the chemotherapy. This could potentially lead to less favorable outcomes, such as in DMFS and DFS. Secondly, as patients with HR- breast cancer do not have adjuvant hormone therapy or targeted therapy to do after surgery, patients with HR- breast cancer should be treated with careful consideration.

Furthermore, considering the increasing rates of pCR, several trials are examining the feasibility of omitting conventional breast surgery following NAC in patients exhibiting pCR as indicated by image-guided vacuum-assisted core biopsy (VACB). In these studies, only patients with negative VACB findings are considered for the elimination of breast surgery, whereas those with residual DCIS are not included [3, 15, 16]. With these approaches, it becomes more important to assess the prognosis in patients with pCR by comparing those with and without residual DCIS.

The increasing importance of NAC in both advanced and early-stage breast cancer is attributed to the rising pCR rates. This study examined the prognostic role of residual DCIS following NAC, considering its significant prevalence in patients who achieved pCR. Although no direct correlation was observed between residual DCIS and breast cancer outcomes in the overall population, remarkably worse outcomes were indicated by the subgroup analysis, especially in patients with HR- breast cancer. To eliminate traditional surgery in patients demonstrating pCR, evaluating prognoses between patients with and without residual DCIS is more important.

The advantages of this study include a large sample size and substantial accumulation of patient-years. This study also has several limitations worth noting. The data were analyzed retrospectively and originated from a single institution, which may constrain the generalizability of our findings.

## Conclusion

In summary, our findings provide a new perspective on the definition of pCR as they demonstrate a divergence in prognosis between patients with and without residual DCIS, particularly in HR- breast cancer. Considering our finding that the prevalence of residual DCIS varies by subtype, more attention should be focused on patients who had residual DCIS after NAC during follow-up compared to patients without residual DCIS, especially in HR- breast cancer. These findings underscore the necessity for further studies involving even larger datasets to validate and analyze this topic.

### Supplementary Information

Supplementary material 1.

## Data Availability

The datasets analyzed during the current study are not publicly available as the personal information of patients must be protected but are available from the corresponding author on reasonable request.
