# Supplementary material for: Association of residual ductal carcinoma in situ with breast cancer treatment outcomes after neoadjuvant chemotherapy according to hormone receptor status
Source: Discov Oncol. 2024 Jul 17;15:288. doi: 10.1007/s12672-024-01157-z (PMC11254890; doi:10.1007/s12672-024-01157-z)
Supplement: Supplementary file 1 — Supplementary material 1. [file 12672_2024_1157_MOESM1_ESM.pdf]

Supplement Figure 1. Kaplan-Meier curves showing DMFS in HER2 status

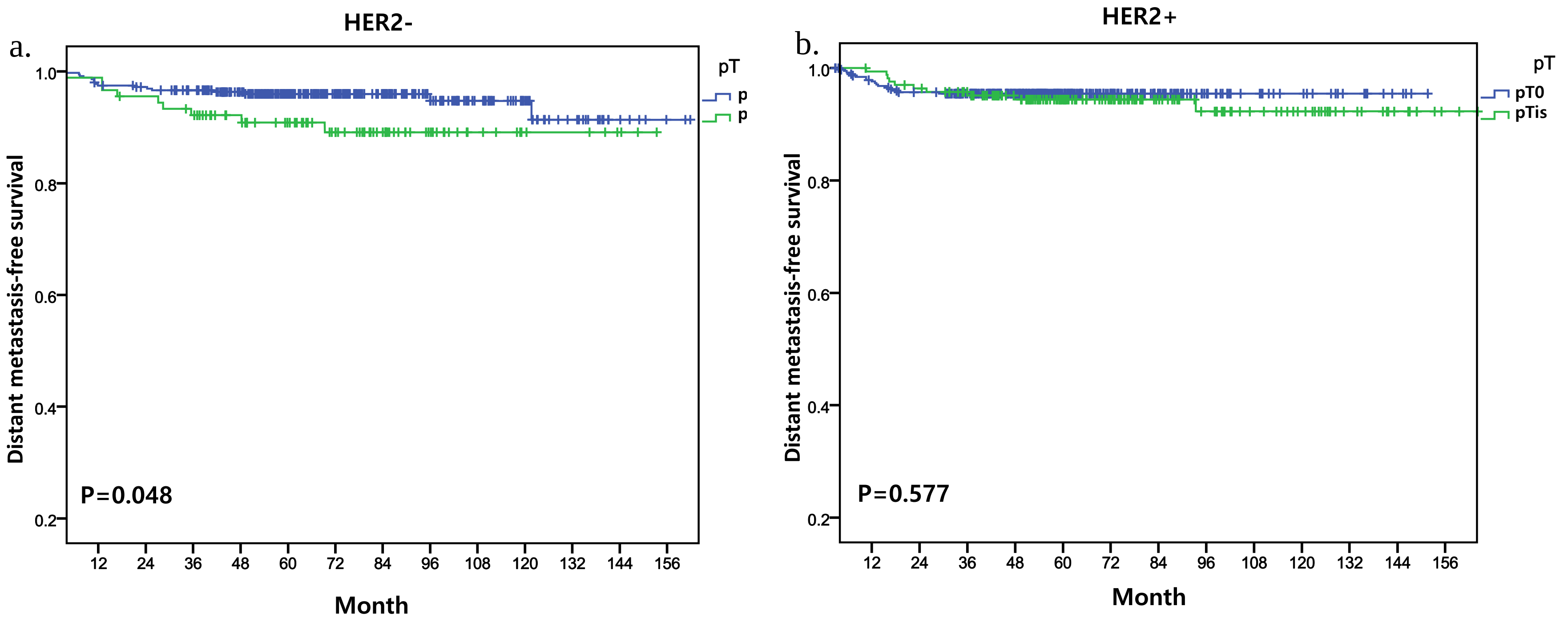

Supplement Figure 2. Kaplan-Meier curves showing LFS and RFS in overall population

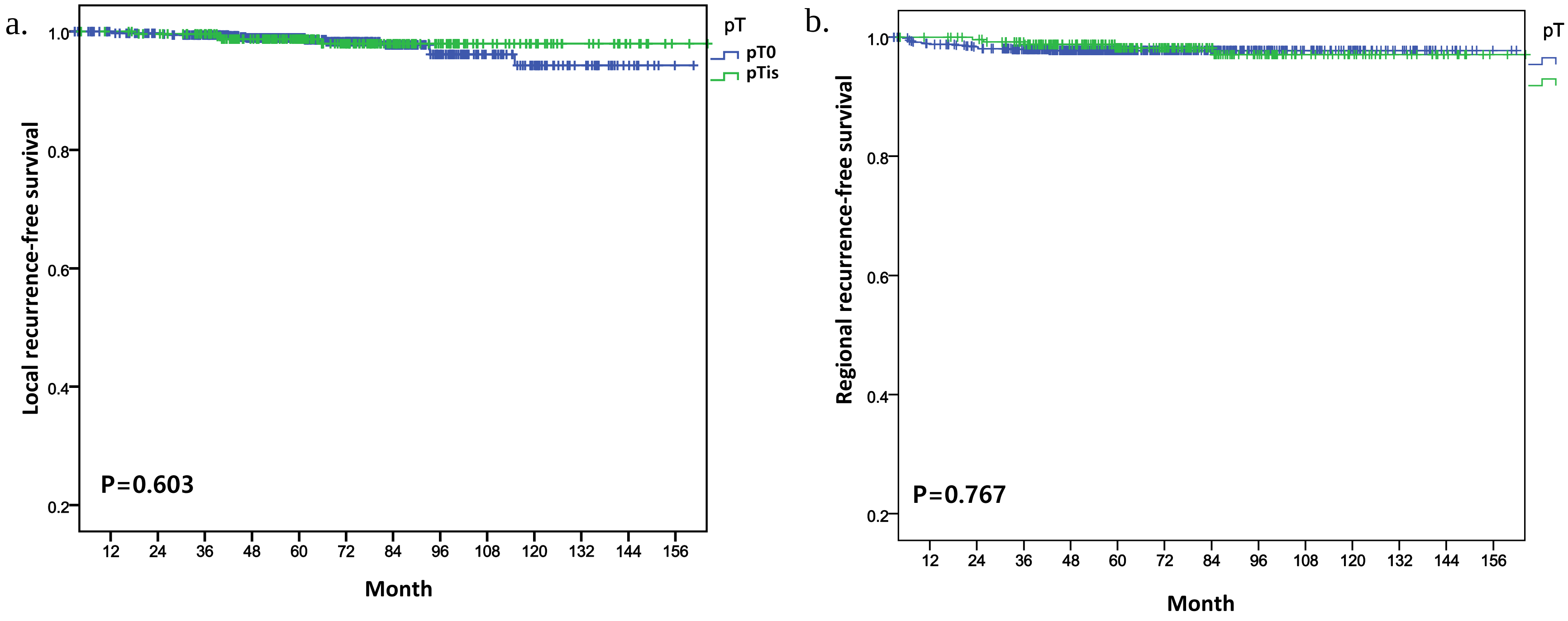

Supplement Figure 3. Kaplan-Meier curves showing LFS and RFS in hormone receptor negative status

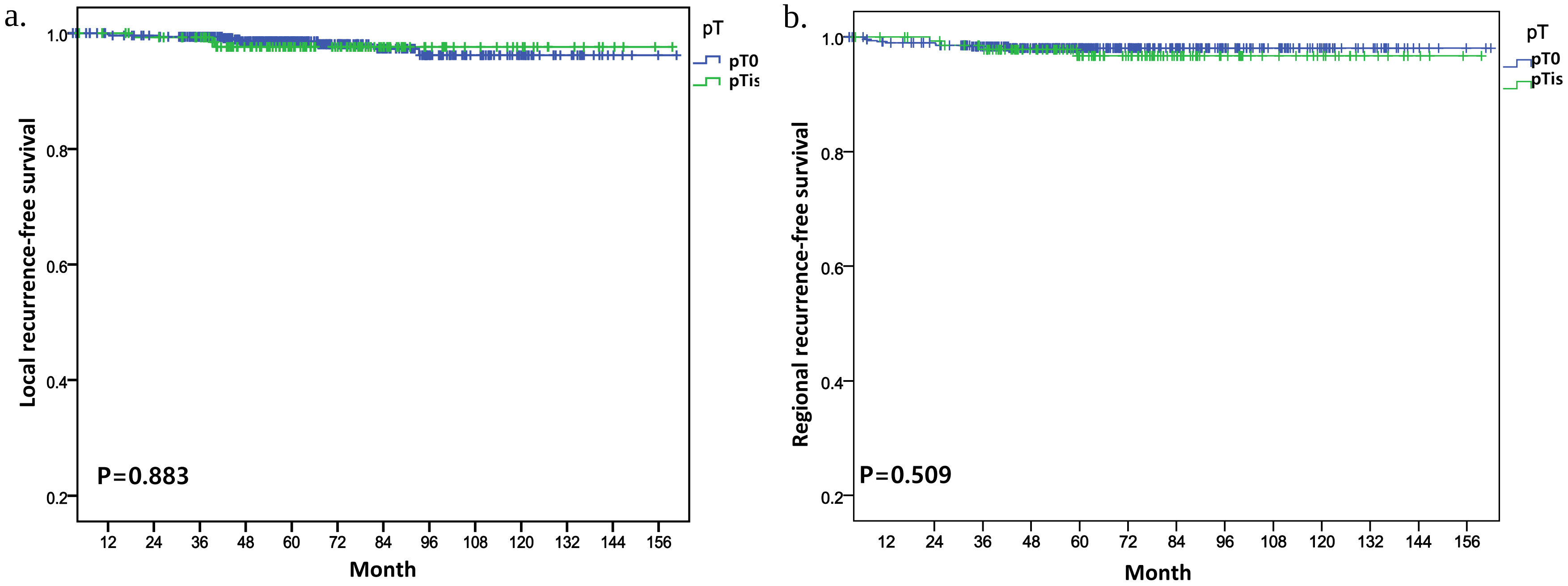

Supplement Figure 4. Kaplan-Meier curves showing LFS and RFS in hormone receptor positive status

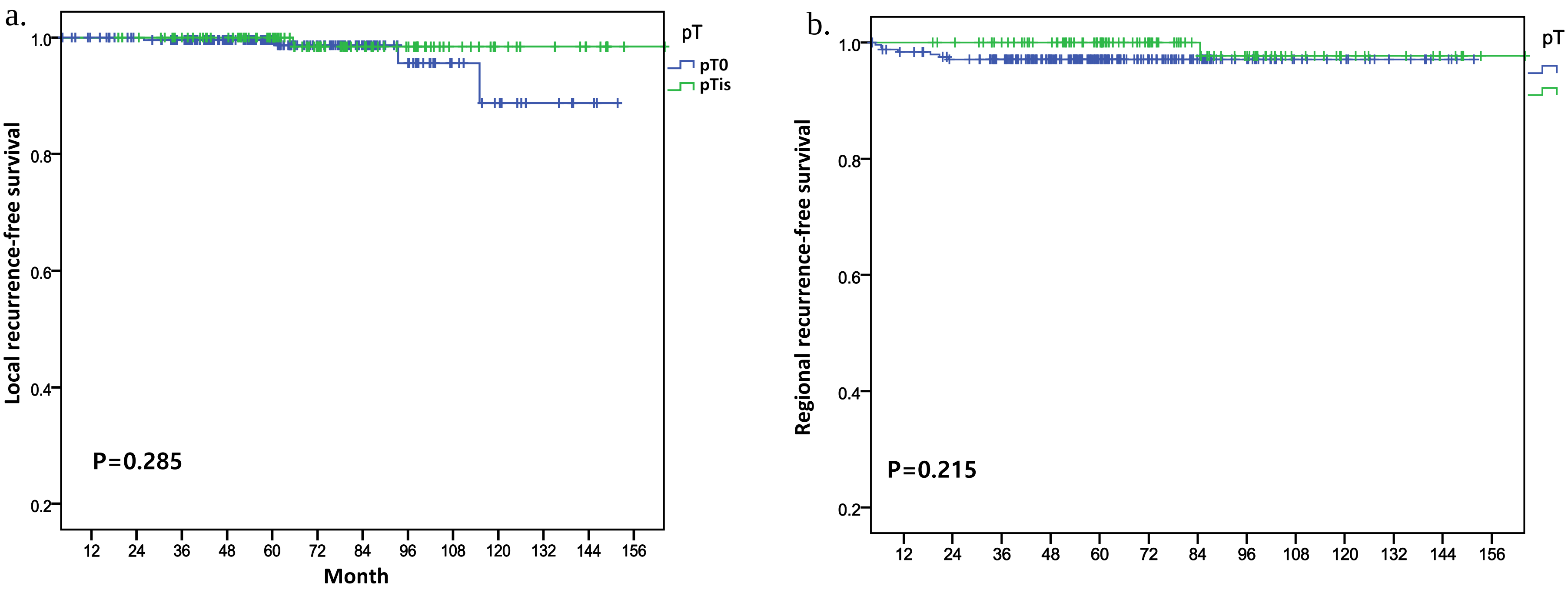

Supplement Table 1. Univariable and multivariable regression analysis in DMFS in overall population

	Univariable		Multivariable	
Variables	HR (95% CI)	P-value	HR (95% CI)	P-value
Age	0.99(0.96-1.01)	0.255	0.99(0.96-1.02)	0.398
Operation		0.075		0.468
Breast conserving surgery	1(Ref)		1(Ref)	
Total Mastectomy	1.64(0.95-2.83)		0.76(0.35-1.62)	
cT		0.810		0.910
cT0-1	1(Ref)		1(Ref)	
cT2-4	0.91(0.41-2.01)		1.05(0.40-2.74)	
cN		0.001		0.010
cN0	1(Ref)		1(Ref)	
cN1	5.66(1.72-18.63)		4.60(1.36-15.53)	
cN2	4.31(0.97-19.28)		2.48(0.41-15.13)	
cN3	7.94(2.31-27.25)		8.14(2.22-29.85)	
Hormone receptor		0.328		0.662
Negative	1(Ref)		1(Ref)	
Positive	1.316		1.17(0.60-2.28)	
HER2 status	0.87(0.56-1.33)	0.514	0.90(0.50-1.62)	0.933
Histologic grade		0.336		0.848
G1-2	1(Ref)		1(Ref)	
G3	0.87(0.63-1.20)		1.40(0.06-32.84)	
Nuclear grade		0.540		0.711
G1-2	1(Ref)		1(Ref)	
G3	0.90(0.63-1.27)		0.54(0.02-12.65)	
p53		0.185		0.271
Negative	1(Ref)		1(Ref)	
Weak	1.23(0.50-3.05)		0.71(0.23-2.21)	
Intermediate	0.82(0.28-2.39)		0.58(0.16-2.04)	
Strong	0.55(0.30-1.02)		0.50(0.25-1.01)	
Ki-67	1.00(0.98-1.01)	0.453	1.00(0.99-1.02)	0.834
Radiotherapy	1.25(0.94-1.66)	0.121	0.61(0.23-1.62)	0.324
Residual DCIS	1.09(0.98-1.23)	0.118	1.32(0.66-2.64)	0.438

HER2 = human epidermal growth factor receptor 2; G = grade

Supplement Table 2. Multivariable analysis of OS after propensity score matching

		pT0	pTis	Hazard Ratio	95% CI		P value
	**Total**	**457**	**238**				
OS	Death	13	16	0.252	0.054	1.171	0.079
	Regional Recurrence	10	5	0.915	0.309	2.710	0.873
	Distant Metastasis	23	17	1.366	0.712	2.621	0.348
	Recurrence	30	19	1.083	0.604	1.944	0.788
	**HR positive status**	**173**	**94**				
OS	Death	5	3	Infinity			
	Regional Recurrence	4	1	0.397	0.054	2.921	0.364
	Distant Metastasis	11	6	0.888	0.327	2.412	0.815
	Recurrence	14	6	0.648	0.262	1.601	0.347
	**HR negative status**	**284**	**144**				
OS	Death	8	13	0.447	0.090	2.208	0.323
	Regional Recurrence	6	4	1.291	0.339	4.921	0.708
	Distant Metastasis	12	11	1.816	0.760	4.337	0.179
	Recurrence	16	13	1.460	0.673	3.164	0.338
